# Chitosan-Boric Acid Scaffolds for Doxorubicin Delivery in the Osteosarcoma Treatment

**DOI:** 10.3390/polym14214753

**Published:** 2022-11-06

**Authors:** Luka Dornjak, Marin Kovačić, Karla Ostojić, Ange Angaits, Joanna Szpunar, Inga Urlić, Anamarija Rogina

**Affiliations:** 1 Faculty of Chemical Engineering and Technology, University of Zagreb, Trg Marka Marulića 19, 10000 Zagreb, Croatia; 2 Faculty of Science, University of Zagreb, Horvatovac 102a, 10000 Zagreb, Croatia; 3 Institute of Analytical and Physical Chemistry for the Environment and Materials (IPREM), UMR 5254, CNRS-University of Pau, Hélioparc, 2, Av. Pr. Angot, 64053 Pau, France

**Keywords:** chitosan, boric acid, doxorubicin, osteosarcoma

## Abstract

Biologically compatible chitosan-based scaffolds have been considered a promising platform for tissue regeneration, tumor treatment, and targeted drug delivery. Chitosan-based scaffolds can be utilized as pH-sensitive drug carriers with targeted drug delivery resulting in less invasive tumor treatments. Further improvement with bioactive ions, such as borate ions, can result in the dual functionality of chitosan carriers provided by simultaneous antitumor efficacy and tissue regeneration. Here, boric acid-containing crosslinked chitosan scaffolds were prepared as delivery systems of doxorubicin, a chemotherapy drug used in the treatment of osteosarcoma. The encapsulation of boric acid was indicated by FTIR spectroscopy, while the ICP-MS analysis indicated the rapid release of boron in phosphate buffer (pH 6.0) and phosphate-buffered saline solution (pH 7.4). The obtained chitosan-boric acid scaffolds exhibit a highly porous and interconnected structure responsible for high swelling capacity, while enzymatic degradation indicated good scaffolds stability during four weeks of incubation at pH 6.0 and 7.4. Furthermore, the release of doxorubicin investigated in phosphate buffers indicated lower doxorubicin concentrations at pH 7.4 with respect to pH 6.0. Finally, the cytotoxicity of prepared doxorubicin-encapsulated scaffolds was evaluated on human sarcoma cells indicating the scaffolds’ potential as cytostatic agents.

## 1. Introduction

Natural human tissue has no mechanism of defense against cancer cells and their rapid replication. One of the primary ways of countering such malicious abnormality is direct cancer removal, chemotherapy, or anti-cancer drugs. Doxorubicin (DOX), a chemotherapy drug, and its bioactive derivatives are among the most widely used drugs for such treatments. Doxorubicin belongs to the anthracyclines class of drugs, together with Daunorubicin, which represents drugs containing aglyconic and sugar moieties used in anti-cancer treatment. DOX is one of the most potent anti-cancer drugs, usually prescribed alone or in synergy with other agents, allowing for a wide spectrum of activity. Usually, it is used in the treatment of solid tumors such as breast, prostate, uterus, stomach, liver, osteosarcoma, and soft tissue sarcomas [1,2]. Anti-cancer properties of DOX are attributed to its ability to intercalate with the DNA double helix and/or binding covalently to proteins associated with DNA replication and transcription. Such interactions ultimately lead to cell apoptosis through DNA, RNA, and protein synthesis inhibition [3,4,5]. In addition to cell apoptosis, high concentrations of DOX may result in permanent damage to the heart, brain, liver, and kidneys, causing cardiotoxicity, lowering cognitive scores, inhibition of self-regeneration, and nephropathy [6,7,8]. However, such treatments lead to multidrug resistance and acute cardiotoxicity, which has led to research into different methods of administrating DOX [9].

The development of less invasive smart materials as drug delivery systems has emerged from the need for advanced procedures of tumor treatment. The targeted drug delivery ensures localized treatment of tumor tissue, minimizing the exposure of the whole body [10]. The potential innovative drug delivery approaches include liposomes, micelles, metal, polymer, or ceramic nanoparticles [11,12,13,14,15], where DOXIL^®^ is one of the clinically available delivery carriers of doxorubicin [16]. Although DOXIL^®^ has been approved, its safety is constantly monitored due to several adverse drug reactions, such as skin reactions and anemia. Nanotechnology has led to numerous studies of potential drug carriers based on metal nanoparticles such as gold, platinum, ruthenium, vanadium, copper, etc. [17], yet with questionable safety due to their accumulation in organs and inappropriate distribution.

Chitosan is known for its biocompatibility, hydrophilicity, non-toxicity, and biodegradability [18,19]. Moreover, chitosan possesses a polycationic nature due to functional amine and hydroxyl groups, which allows pH-dependent drug release. Its hydrogel nature offers great encapsulation capacity for biomolecules, ions, and drugs while possessing structural properties similar to the extracellular matrix (ECM) for less disruptive implementation and treatment [20,21]. Chitosan-based drug delivery systems were prepared as pH-responsive nanoparticles or nanocapsules [22,23,24] to provide intracellular delivery of antitumor drugs. 

The important issue that needs to be addressed is the regeneration of treated and surrounding tissue after tumor resection and postoperative chemotherapy cycles. Normally, bone regeneration can be induced by growth factors such as BMP-2 and BMP-7 (bone morphogenic proteins), approved by Food and Drug Agency in 2002. However, growth factor therapy is controversial in malignant disease treatment since it can potentially stimulate the growth of tumor cells and metastasis [25]. Such restraints in clinical practice brought forth alternative strategies for tissue regeneration by using bioactive agents, such as boron. Boron plays a significant role in the healthy metabolism of bone cells and vascularization essential for the formation of new tissue [26]. Recently, boron has been recognized as an element predominant for the homeostasis of the human body [27].

In this work, we propose chitosan-based scaffolds as potential drug delivery systems of DOX to treat tumor residues after surgical resection of the tumor. The successful encapsulation of DOX was achieved by genipin crosslinking reactions with chitosan while preserving the highly porous and interconnected structure of the scaffold-based drug delivery system. To extend the functionality of drug carriers, scaffolds were simultaneously modified by boric acid as a source of boron, a bioactive agent, for providing the angiogenic potential of prepared scaffolds. Proposed chitosan-based scaffolds were developed as potential support for tumor inhibition, and tissue regeneration in defects originated from surgical resection of the tumor.

## 2. Materials and Methods

Chitosan (CHT, Chitoscience 85/200, product number 23,505) with a degree of deacetylation (DD) of 83% and viscosity of 293 mPa was purchased from Heppe Medical Chitosan, HMC^+^ (Halle, Germany). Acetic acid (99.8%) and boric acid (p.a.) were purchased from Lachner (Neratovice, Czech Republic), doxorubicin HCl was purchased from Carbosynth Ltd. (Berkshire, UK), acetone (p.a.) was purchased from T.T.T (Sveta Nedjelja, Croatia), and genipin was purchased from Cayman Chemical Company (Ann Arbor, MI, USA). 

### 2.1. Synthesis of Boric-Acid-Modified Chitosan Scaffolds

Chitosan-boric acid scaffolds were prepared as follows: CHT was dissolved in acetic acid 0.5% (*v*/*v*) to obtain a clear 1.2% (*w*/*v*) chitosan solution. Then, different aliquots of boric acid (BA) solution were added to the CHT solution to obtain a final BA concentration of 10 and 20 mmol/dm^3^. After 24 h of stirring, genipin (a crosslinker) was added (2% *w*/*w* with respect to chitosan), and stirring was continued for 4 h. After homogenization, the obtained solutions were cast into a 24-well plate and incubated at 50 °C for 24 h for crosslinking reactions. Crosslinked hydrogels were then frozen at −22 °C and lyophilized for 48 h using a Kambic LIO-5PLT freeze-dryer. The dried scaffolds were washed in acetone to remove any residues of unreacted genipin. The samples were denoted according to the BA concentration as CHT BA0, CHT BA10, and CHT BA20 in crosslinked gels.

### 2.2. Encapsulation of Doxorubicin into Chitosan-Boric Acid Scaffolds

Boric-acid-modified chitosan scaffolds were prepared as a drug delivery system for the local administration of DOX. The encapsulation of DOX was conducted as follows: the chitosan-boric acid solution was prepared as previously described, and different aliquots of DOX dissolved in demineralized water (concentration of 0–3.7 mg/mL) were added under intensive stirring for 2 h in order to obtain final DOX concentration of 0–100 μg/mL in polymer solution. Then, genipin was used to crosslink the prepared CHT BA DOX solution as previously described. Crosslinked hydrogels were frozen at −22 °C and lyophilized for 48 h. The dried scaffolds were then washed in acetone to remove any crosslinker residues.

### 2.3. Scaffolds Identification

The composition of chitosan-boric acid scaffolds was analyzed by FTIR spectroscopy using Bruker Vertex 70 ATR-FTIR spectrometer at 20 °C, at a resolution of 2 cm^−1^ and a spectral range of 4000–650 cm^−1^ with 32 scans.

### 2.4. Scaffolds Characterization

The release of boron was evaluated in phosphate buffer (PB, pH 6.0) and phosphate-buffered saline solution (PBS, pH 7.4) for 24 h at a temperature of 37 °C. At predetermined time, the supernatants were collected, filtered, and analyzed using ICP-MS. The ICP-MS analysis was carried out by using a reaction cell pressurized with He and H_2_ gas. The isotopes monitored were 10B and 11B. Analytical blanks were analyzed in parallel. The Standard Reference Material 1643. L1 Trace metals in water (CPAchem Ltd., 2, Bogomilovo, Bulgaria) was used for quality control. Samples were diluted 5000-fold in 2% HNO_3_. Quantification was performed in the calibration range of 0.05–10 ppb. Measurements were carried out in triplicate; the results with a relative standard deviation higher than 10% were discarded, and the measurements were repeated. Values are reported as mean ± standard deviation (SD) of three analytical replicates.

The microstructure of chitosan-boric acid scaffolds was investigated by scanning electron microscope (SEM) TESCAN Vega3SEM Easyprobe with the electron beam energy of 10 keV. Prior to imaging, samples were sputtered with gold and palladium. 

### 2.5. In Vitro Degradation of Chitosan-Boric Acid Scaffolds

The degradation and swelling capacity of chitosan-boric acid scaffolds were estimated in phosphate buffer (PB, pH 6.0) and phosphate-buffered saline solution (PBS, pH 7.4) supplemented by lysozyme (LYS, 1 mg/mL) during four weeks of incubation in an orbital shaker at 50 rpm and temperature of 37 °C. The incubation medium was replaced by a fresh solution every third day. To avoid bacteria and algae growth, sodium azide was added to the buffer solutions (0.2 mg/mL). At predetermined time, samples were carefully collected from the medium, washed with demineralized water three times, and weighed to determine swelling capacity. Then, wet samples were dried at 40 °C until constant mass. The degradation percentage was expressed as a weight loss compared to the initial weight of the samples.

### 2.6. In Vitro Drug Release Study

The release of DOX was evaluated in phosphate buffers with different pH (6.0 and 7.4) for 24 h at a temperature of 37 °C. At predetermined time, the supernatants were collected and analyzed using HPLC. A Shimadzu LC-20 HPLC system consisting of a SIL-10AF autosampler, binary LC-20 pumps with an integrated system controller, DGU-20A5R degasser, CTO-20A column thermostat, and SPD-M20A UV/DAD detector was used to determine the concentration of DOX in solution. Chromatographic separation of DOX was performed on a Nucleosil 120-5 C18 RP column (250 mm × 4.6 mm, 5 μm particle size) using isocratic elution. The mobile phase consisted of a 40:60 *v*/*v* aqueous (0.5 % *v*/*v* formic acid and 5% *v*/*v* MeOH):MeOH phase at a total flow rate of 1 mL/min. The sample injection volume was 100 μL. The column temperature was maintained at 35 °C. DOX was quantified at a wavelength of 480 nm and at a retention time of 13.1 min.

### 2.7. Cell Viability Assay

The cytotoxic effect of DOX-encapsulated chitosan-boric acid scaffolds was evaluated on U2OS cells using the 3-(4,5-dimethylthiazol-2-yl)-2,5-diphenyltetrazolium bromide (MTT) assay. 

U2OS cells were cultured in Dulbecco’s modified Eagle medium with 4500 mg/L glucose (DMEM-high glucose; Capricorn Scientific) supplemented with 10% fetal bovine serum (FBS; Sigma-Aldrich, Burlington, MA, USA) and 1% penicillin/streptomycin (Sigma-Aldrich, Burlington, MA, USA). When they had reached 80% confluence, they were seeded into each well of a 24-well plate (Sarsted) in a concentration of 5 × 10^4^ cells/2 mL of the medium and allowed to adhere overnight in a humidified incubator with 5% CO_2_ at 37 °C.

On the second day, the medium was removed, and cells were incubated with media containing DOX-encapsulated scaffolds and free DOX at different concentrations for 72 h. Each sample was tested in triplicate.

Following the incubation period, the medium was removed, and cells were treated with 100 μL/well of MTT solubilized in cell medium at 0.5 mg/mL concentration. After 3.5 h of incubation, 500 μL dimethyl sulfoxide (DMSO, Sigma-Aldrich, Burlington, MA, USA) was added to each well to dissolve formed crystals (15 min). After 15 min of dissolving, absorbance was measured at 560 nm using the microplate reader (Glomax-Multi, Promega). Cell viability was calculated as a percentage of untreated cells (negative control).

## 3. Results and Discussion

### 3.1. FTIR Analysis

**Figure 1:**
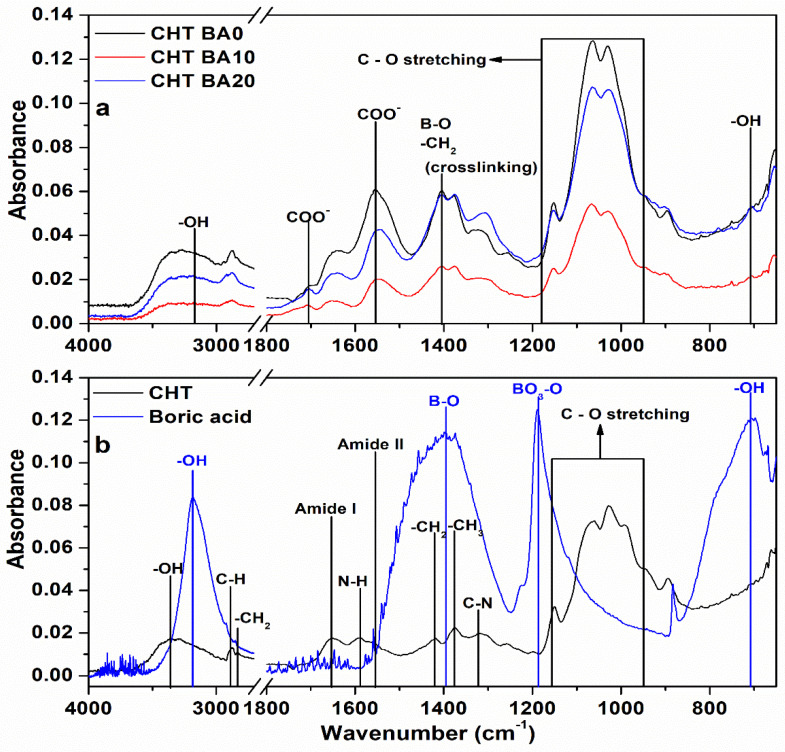
FTIR spectra of (**a**) boric acid–modified chitosan scaffolds and (**b**) pristine chitosan scaffolds and boric acid.

ATR-FTIR spectroscopy was used to identify the composition of prepared chitosan-boric acid scaffolds. As seen in Figure 1, pristine chitosan shows characteristic absorption bands: the band overlap in the region between 3360 and 3289 cm^−1^, which corresponds to stretching vibrations of the hydroxyl groups and amino and their interactions through H-bond; two absorption bands at 2873 and 2838 cm^−1^ can be attributed to symmetric and asymmetric stretching of C–H in –CH_2_ group; three absorption bands at 1651, 1589, and 1557 cm^−1^ that can be associated with stretching vibration of carbonyl group in amide (amide I), vibration of N–H in primary amines; and N–H bending in combination with C–N stretching vibrations (amide II), respectively [28,29,30]. The absorption bands at around 1419, 1375, and 1315 cm^−1^ can be associated with bending vibrations of –CH_2_ from the pyranose ring and –CH_3_ groups and stretching vibrations of the C–N bond (amide III), while the band at 1150 cm^−1^ can correspond to asymmetric stretching of C–O–C bridge. Two absorption bands at 1061 and 1027 cm^−1^ can be attributed to the stretching of C–O bonds [29,31,32,33,34,35,36].

Significant changes in FTIR spectra were observed in crosslinked scaffolds with respect to the pristine chitosan. The new band at ~1705 cm^−1^ was observed in all CHT BA samples, which could correspond to the carboxylic acid group from acetic acid used as a solvent for scaffold preparation [37]. Furthermore, the increased intensity and shift of the absorption band at 1547–1542 cm^−1^ could be attributed to the carboxylic group [37], and the amide II band of secondary amide formed due to the reaction between the ester and hydroxyl groups of genipin and amino groups of chitosan [29]. Furthermore, the shift of the amide I band from 1651 to 1644–1641 cm^−1^, the absorption band at 1405 cm^−1^, and the increased intensity of the absorption band at 1065 cm^−1^ could be a result of chitosan-genipin crosslink [29]. 

The spectrum of boric acid exhibits characteristic absorption bands at 3184, 1400, and 705 cm^−1^, which are attributed to the stretching vibrations of the O–H bond, asymmetric stretching of the B–O bond, and stretching vibrations of the –OH bond of trigonal boric acid, respectively [38,39,40,41]. The presence of boric acid in CHT BA scaffolds could be observed by broadening of region between 3360 and 3100 cm^−1^, which corresponds to band overlap associated with amino and hydroxyl groups from chitosan and the hydroxyl group of boric acid. Furthermore, the absorption band at 705 cm^−1^ has shifted to higher wavenumbers with respect to that of pure boric acid indicating weak interactions with the polymer matrix. The absorption band at 1409 cm^−1^ was difficult to observe due to the overlap of the absorption band of genipin. 

Boric acid has been used as a crosslinker for hydroxyl acids and polyol polymers, where, depending on the pH of the solution, neutral boric acid or negatively charged borate ions form complexes with hydroxyl groups [42,43,44]. A recent study [45] on graphene oxide-chitosan film proposed crosslinking reaction between organic and inorganic components through borate ions. The authors reported on possible contribution of the boron-amine complexes along with the reactions of tetrahydroxyborate ions (B(OH)_4_^−^) and hydroxyl groups of chitosan molecules. Another study [46] also suggested the formation of a chitosan-borate complex, while Uddin et al. [47] proposed the possibility of intermolecular hydrogen bonding. Our results might suggest the formation of weak interactions between the boric acid and chitosan-genipin matrix.

### 3.2. Boron Release

**Figure 2:**
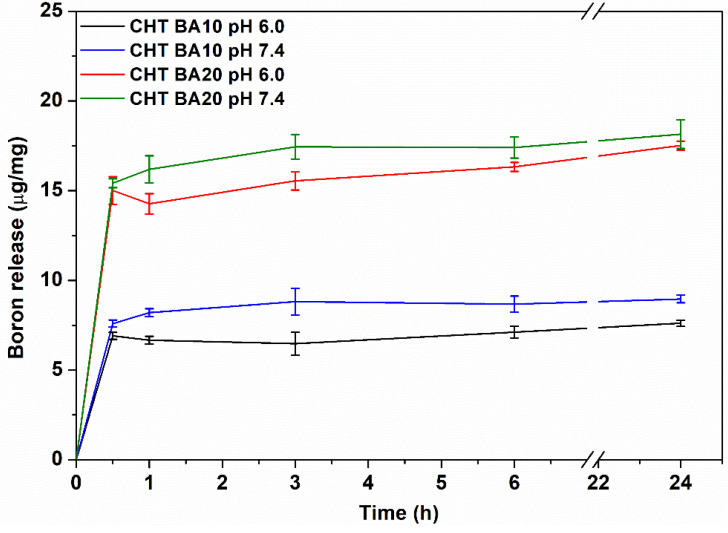
Boron release in PB (pH 6.0) and PBS solution (pH 7.4) at 37 °C.

The release profile of boron from chitosan-boric acid scaffolds is shown in Figure 2. Both CHT BA scaffolds showed rapid boron release when incubated in PB and PBS solutions, with B quantity of approximately 7.5 and 15 μg/mg for CHT BA10 and CHT BA20, respectively. Similar values of B released from the scaffolds were kept after 24 h of incubation, indicating the equilibrium immediately after scaffold incubation in buffer solutions. This observation could indicate weak interactions between chitosan functional groups and boric acid. Similar findings were reported by Rico et al. on borax-loaded PLLA films [26], where the total amount of boron was released within 3 h in Dulbecco’s PBS solution. The impact of different pH of buffer solution was observed by slightly higher released B values at pH 7.4; however, the observed difference was not considered as significant. The rapid release of B from the scaffolds might also be facilitated by the high swelling capacity characteristic of chitosan-based scaffolds. As a therapeutic agent, boron is usually incorporated into bioactive glasses to modulate angiogenesis and neovascularization and to have a slower controlled release from the materials in the physiological microenvironment [48]. Recent studies on synthetic and natural polymers modified by borax and boric acid as boron precursors indicated enhanced vascularization and angiogenesis provided by rapid or moderate boron delivery at lower concentrations [26,40]. Based on the observed fast B release from CHT BA scaffolds, proposed materials could show angiogenic potential, which will be investigated in future studies.

### 3.3. Scaffold Microstructure

**Figure 3:**
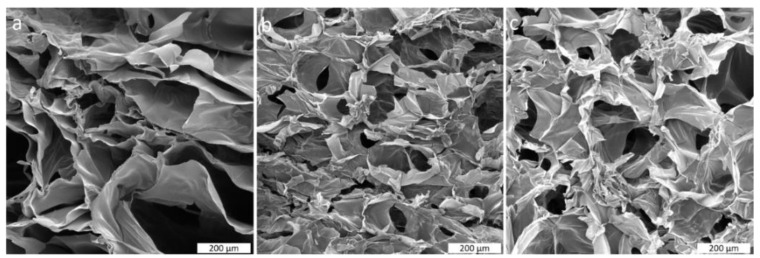
SEM micrograph of (**a**) CHT BA0, (**b**) CHT BA10, and (**c**) CHT BA20 scaffolds. Scale bar: 200 μm.

The microstructure of chitosan-boric acid scaffolds is characterized by high porosity and interconnected pores of irregular shape, as shown in Figure 3. Chitosan-genipin scaffolds are mostly prepared to improve mechanical properties, such as stiffness that enhances cell adhesion and proliferation with respect to pristine chitosan scaffolds [49]. In this work, genipin was used to incorporate boric acid and DOX into the chitosan scaffold and preserve them during the scaffold preparation protocol. The lyophilization of prepared chitosan-genipin hydrogels gave macroporous scaffolds with visually assessed pore sizes up to 200 μm. The high porosity of prepared scaffolds is also favorable for faster delivery of therapeutics and drugs due to higher absorption capacity. Furthermore, proposed scaffolds should also serve as temporary support for tissue regeneration, where porosity and pore size are predominant factors for neovascularization and new tissue ingrowth. According to Harish Prashanth et al. [50], the optimal pore size for cells, nutrient, and waste migration is ~100 µm; therefore, prepared scaffolds could be suitable candidates for cell adhesion and growth. The addition of boric acid did not significantly affect the porosity of the scaffolds while resulting in a more uniform porous structure. 

### 3.4. Degradation Behavior

**Figure 4:**
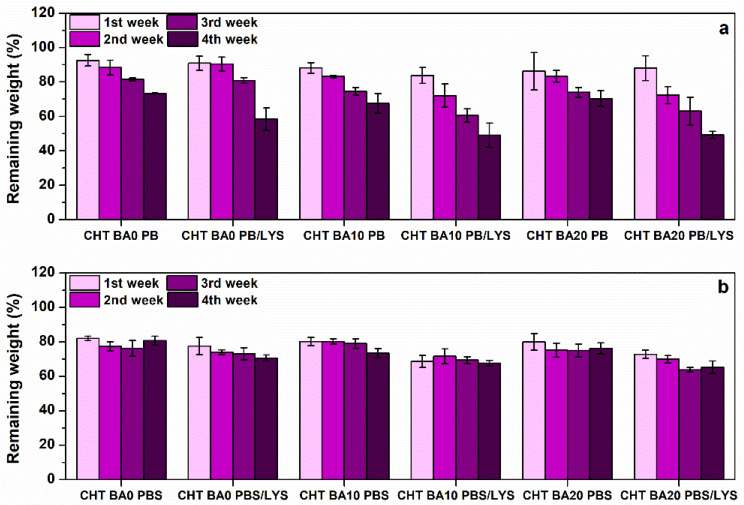
Enzymatic degradation of chitosan-boric acid scaffolds in (**a**) phosphate buffer (pH 6.0) and (**b**) phosphate buffered saline solution (pH 7.4) with and without lysozyme.

The degradation behavior of chitosan-boric acid scaffolds was estimated in PB and PBS solution in the presence of lysozyme (LYS) during 4 weeks of incubation (Figure 4). The scaffolds incubation in PB and PBS without lysozyme served as a control. The degradation of scaffolds in PB solution (pH 6.0) was higher during 4 weeks with respect to the scaffolds incubated in PBS (pH 7.4), with gradual weight loss during the incubation period. The scaffolds incubated in PB/LYS solution showed drastic weight loss between approximately 40 and 55% after 4 weeks of lysozyme activity. Specifically, the boric-acid-modified scaffolds exhibited lower remaining weight after 4 weeks of incubation with respect to the chitosan scaffold. Significant changes in weight loss during incubation in PB solution without lysozyme were not observed between the scaffolds. Still, all systems showed weight loss of approximately 30% after 4 weeks in PB solution. According to the previous works on chitosan-genipin scaffolds [51,52], the crosslinking degree reaches a certain value and remains similar even at higher genipin concentrations. This means that not all amine groups of chitosan are involved in crosslinking reactions and can be protonated in acidic solutions. Possible dissolution of chitosan was expected at this pH value since the p*K* of chitosan is around 6.3–6.5 (depending on deacetylation degree).

The degradation test in PBS, both with and without lysozyme activity, indicated higher stability of the scaffolds. All scaffolds show initial weight loss of approximately 20–30% after the first week in PBS/LYS and PBS solution, which was followed by a slower degradation rate. After 4 weeks in PBS/LYS, all systems showed degradation of approximately 30–35%, whereas chitosan-boric acid scaffold CHT BA20 showed slightly higher weight loss. As expected, samples incubated in PBS/LYS exhibited higher weight loss with respect to scaffolds incubated in PBS, which can be a result of lysozyme-induced degradation.

**Figure A1:**
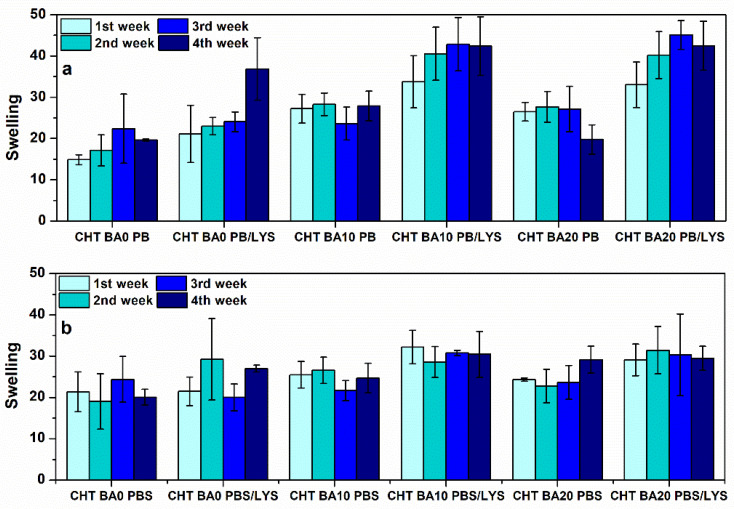
Swelling capacity of chitosan-boric acid scaffolds in (**a**) phosphate buffer (pH 6.0) and (**b**) phosphate-buffered saline solution (pH 7.4) with and without lysozyme.

The swelling ratio of degraded scaffolds was also estimated during 4 weeks of incubation (Appendix A, Figure A1). All systems exhibited a high swelling ratio (above 20) which is a result of the chitosan hydrogel nature and porosity accompanied by scaffolds dissolution and degradation. After 4 weeks, chitosan-boric acid scaffolds incubated in PB/LYS solution indicated a higher swelling ratio between 37 and 42 with respect to the samples incubated in PB solution (swelling ratio of 20–27). During degradation, the scaffold’s structure changes from stable to disintegrated, with larger voids between the pores [53]. Furthermore, the pore size increases with degradation time, allowing for higher medium absorption, i.e., increased swelling capacity. Based on the higher weight loss of scaffolds after 4 weeks of incubation, the higher swelling ratio could be a result of increased porosity. On the other hand, samples incubated in PBS/LYS and PBS solution did not show a significant difference in swelling capacity during 4 weeks of degradation, with a swelling ratio between 20 and 30. The biodegradation rate is an important property of potential tissue substituent and should correlate with the rate of new tissue ingrowth by serving as temporary support for cells. Based on estimated weight loss, the proposed scaffolds show good stability under enzymatic degradation.

### 3.5. Doxorubicin Release

**Figure 5:**
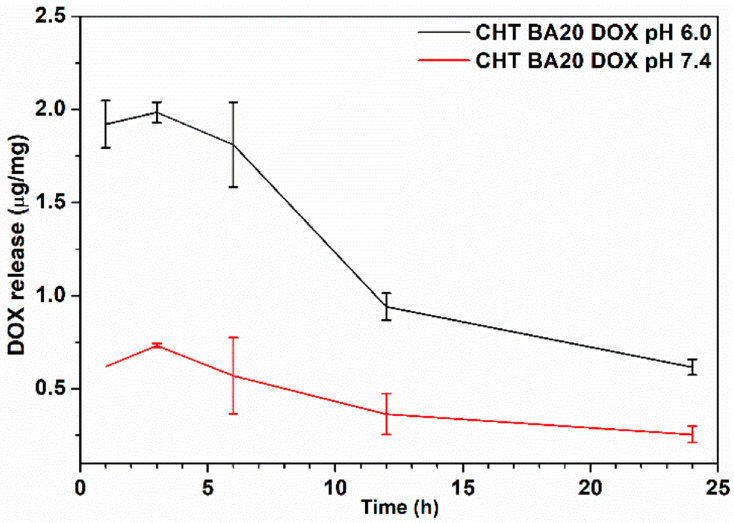
In vitro release of DOX from CHT BA scaffold in PB with different pH at 37 °C.

**Figure A2:**
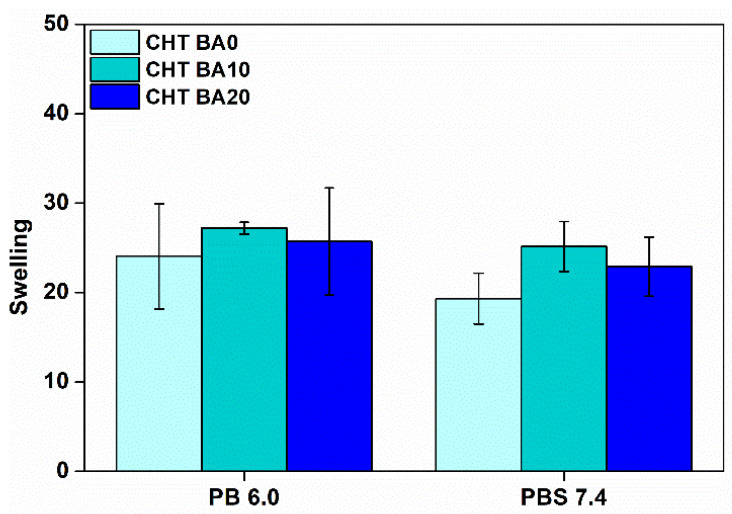
Swelling capacity of chitosan-boric acid scaffolds in buffer solutions with different pH (PB 6.0, PBS 7.4) after 24 h.

The doxorubicin release profiles from chitosan-boric acid scaffolds were evaluated in phosphate-buffer (PB) solution with different pH (pH 6.0 and 7.4) for 24 h (Figure 5). At pH 6.0, the amount of DOX released from the scaffold during the first 6 h was similar, indicating rapid release of the drug due to the high swelling ability of the drug carrier (Appendix A, Figure A2). Observed behavior is expected for chitosan-based carriers, according to previous studies [22,23,54,55]. 

After 12 h, a drop in DOX amount of approximately 50% was observed without replacing the medium with fresh buffer solution during the release study. The DOX amount continued to decrease after 24 h with a value of only approximately 30% of the initially released amount. A similar trend was observed during the release study in phosphate buffer pH 7.4, with lower initially released concentrations. The drop in DOX concentration after 6 h could be attributed to DOX hydrolysis (primarily) and self-dimerization (secondary stage) under acidic and alkaline conditions [56], [3]. According to Yamada [3], Fülöp et al. [57], and Menozzi et al. [58], aqueous solutions of DOX undergo precipitation in buffer media. Yamada [3] investigated temperature- and pH-dependent DOX precipitation in phosphate buffers and showed larger precipitation at higher pH caused by DOX dimerization. The author observed ~50% of DOX precipitation at pH 6.0 and ~90% at pH 7 after 24 h at 37 °C. Our results showed that the released DOX amount in PB pH 7.4 was approximately 70% lower than the ones released in PB pH 6.0 at 37 °C, which could be a result of mentioned DOX precipitation. 

Previous studies on chitosan-based delivery systems of DOX [22,23,54,55] proposed that chitosan’s polycationic nature was responsible for higher DOX release under acidic conditions, which are characteristic of the tumor microenvironment. Taking into account similar swelling behavior of CHT BA scaffolds at pH 6.0 and 7.4 (Appendix A, Figure A2), our findings suggest that DOX stability, its hydrolysis, and dimerization are predominant factors that cause a difference in DOX release under acidic and neutral (i.e., physiological) conditions. Furthermore, it has been shown that the products of DOX dimerization [3,59] exhibit weak cytotoxic activity depending on the dosage and the cell type. Still, doxorubicin finds its clinical application in the treatment of several tumors. 

### 3.6. Cytotoxicity Assay

**Figure 6:**
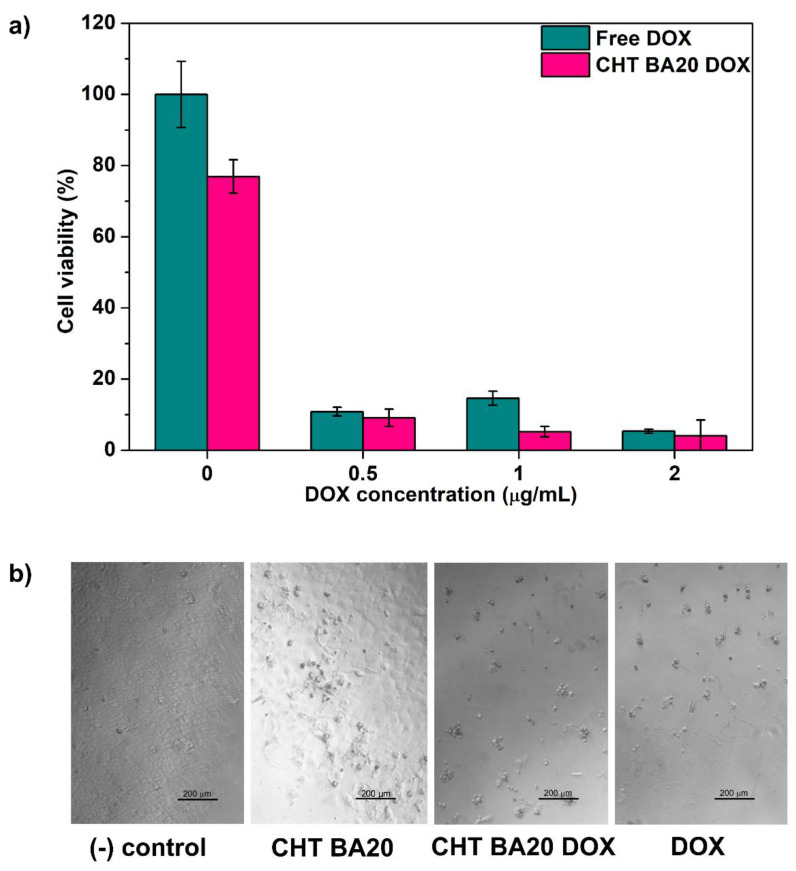
(**a**) Cell viability assay of U2OS cells cultured with DOX-encapsulated chitosan-boric acid scaffolds for 3 days; (**b**) Optical micrographs of U2OS after 3 days of incubation (scale bar: 200 µm).

Human sarcoma cells, U2OS, were employed to investigate the cytotoxic activity of DOX-encapsulated chitosan-boric acid scaffold, while free DOX was used as a positive control (Figure 6) 80% of cell viability compared to nontreated cells (negative control) was considered as the cytotoxic limit [60,61]. The results indicated that chitosan-boric acid scaffolds have no cytotoxic effect when directly incubated with the tumor cells. On the other hand, a dramatic reduction of viability was observed in tumor cells incubated with DOX-encapsulated scaffold at different drug dosages as a result of DOX release. Optical micrographs of U2OS cells cultured in direct contact with materials indicated spherical morphology of cells when DOX was present, implying cell death. Taking into consideration DOX hydrolysis and possible dimerization during the first hours of release, proposed scaffolds have the potential to be further studied on patient-specific tumor cells. 

## 4. Conclusions

This work proposes novel DOX-encapsulated chitosan-boric acid scaffolds as potential delivery systems of therapeutic agents and antitumor drugs. Prepared scaffolds showed simultaneous fast delivery of boron and DOX, which might provide dual functionality of the material. Furthermore, our data indicated doxorubicin hydrolysis and possible dimerization during the release study in phosphate buffers, which gives a conclusion opposite to previous studies on pH-responsive DOX-chitosan-based delivery systems. We assume that lower DOX concentrations measured at physiological pH with respect to mildly acidic are mainly caused by DOX hydrolysis and dimerization. Still, the proposed materials showed cytotoxicity toward human sarcoma cells after 3 days of incubation.

## Data Availability

The datasets used and analyzed during this study are available from the corresponding author upon reasonable request.
